# A pilot randomized controlled trial of the iPeer2Peer program in adolescents with sickle cell disease: A mixed method study

**DOI:** 10.1017/cts.2024.1170

**Published:** 2025-01-03

**Authors:** Lauren Kelenc, Brittany Wiles, Fareha Nishat, Chitra Lalloo, Anya Nair, Craig Eling, Melanie Kirby-Allen, Ewurabena Simpson, Roona Sinha, Richard Ward, William T. Zempsky, Sara Ahola Kohut, Jennifer N. Stinson

**Affiliations:** 1 Child Health Evaluative Sciences, The Hospital for Sick Children Research Institute, Toronto, Canada; 2 Jim Pattison’s Children’s Hospital, Saskatoon, Saskatchewan, Canada; 3 Division of Haematology/Oncology, The Hospital for Sick Children, Toronto, Ontario, Canada; 4 Division of Haematology/Oncology, Children’s Hospital of Eastern Ontario, Ottawa, Ontario, Canada; 5 Toronto General Hospital, University Health Network, Toronto, Ontario, Canada; 6 Department of Medicine, Temerty Faculty of Medicine, University of Toronto, Toronto, Ontario, Canada; 7 Division of Pain & Palliative Medicine, Connecticut Children’s Medical Center, Hartford, CT, USA; 8 Department of Pediatrics, University of Connecticut School of Medicine, Farmington, CT, USA; 9 Division of Gastroenterology Hepatology and Nutrition, The Hospital for Sick Children, Toronto, Ontario, Canada; 10 Lawrence S Bloomberg Faculty of Nursing, University of Toronto, Toronto, Canada

**Keywords:** Online, peer support, feasibility, adolescent, sickle cell disease, self-management

## Abstract

**Background and objectives:**

Sickle cell disease (SCD) is hallmarked by recurrent episodes of severe acute pain and the risk for chronic pain. Remote peer support programs have been shown to effectively improve health outcomes for many chronic conditions. The objective of this study was to examine the feasibility and acceptability of an online peer mentoring program (iPeer2Peer program) for adolescents with SCD.

**Method:**

A waitlist pilot randomized controlled trial was conducted. Adolescents randomized to the intervention group were matched with trained peer mentors (19–25 years; successfully managing their SCD), consisting of up to 10 sessions of approximately 30-min video calls over a 15-week period. The control group received standard care. The primary outcomes were rates of accrual, withdrawal, and adherence to iP2P program/protocol, with secondary outcomes identifying topics of mentorship–mentee conversations through qualitative analysis.

**Results:**

Twenty-eight participants (14 intervention; 14 control) were randomized to the study (mean age: 14.8 ± 1.7 years; 57% female). Accrual rate was 80% (28/35) and withdrawal rate was 18% (5/28), with 28% (4/14) adhering to the iP2P program; however, 71% (10/14) of adolescents in the intervention completed at least one call. Based on content analysis of 75 mentor–mentee calls, three distinct content categories emerged: impact of SCD, self-management, transitioning to adulthood with SCD, and general topics.

**Conclusion:**

The results from this pilot study suggest that the current iteration of the iP2P SCD program lacks feasibility. Future research with the iP2P program can focus improved engagement via personalized mentoring, variable communication avenues, and an emphasis on gender.

## Introduction

Sickle cell disease (SCD) is the most common genetic blood disease in North America and primarily affects people of African descent [1,2]. The hallmark feature of SCD is recurrent episodes of acute severe pain due to vaso-occlusion events (VOEs; blockage of red blood cells) [1]. SCD pain is reportedly worse than postoperative pain, as intense as terminal cancer pain, and has a negative impact on all aspects of health-related quality of life (HRQL) [3–5]. Pain burden of SCD increases as adolescents grow from childhood to young adulthood [6]. In addition to acute pain, many adolescents with SCD also experience daily chronic pain [7]. This pain has negative consequences on physical and mental well-being (e.g., depression and anxiety), and daily life activities including academic underachievement related to missing school, little or no opportunities for social interaction with peers, poor sleep, and high stress [8,9].

Nearly 90% of SCD pain episodes are treated in the home setting [10]. Unfortunately, many of these episodes may not be optimally managed due to a lack of knowledge, self-advocacy, and social support [5,10]. As a result, adolescents with chronic conditions face barriers from their disease such as lack of attendance at school and social events, and demanding treatment regimens [11]. For SCD, prevalent among marginalized and underrepresented communities [12], socially based interventions show promise as they can provide social support to improve self-management skills [4,13].

Peer support in healthcare is an explicit form of social support established to provide individuals with emotional (e.g., expressions of caring, empathy, and reassurance), appraisal (e.g., affirmation of one’s feelings and behaviors, encouraging persistence for resolving problems and reassurance that frustrations can be handled), and informational (e.g., providing advice, suggestions, and facts relevant to issues with which the peer is dealing) support by another person living with a similar condition [14]. The provision of these attributes has been associated with improved health outcomes among those with chronic conditions in adult populations; however, less is known about its application in pediatric populations [15,16]. Therefore, there is a need to examine peer mentoring as a means to improve social support in pediatric populations, specifically through randomized controlled trials (RCTs) [16–18].

There is increasing interest in remote support because of greater accessibility and convenience it offers when compared to in-person interactions. The literature suggests that peer support programs delivered remotely can effectively improve health outcomes for many conditions [19–21]. Remote support provides patients and their families with opportunities to receive helpful social support and gain access to evidence-based health information to better manage their disease, while simultaneously tackling historical systemic, geographical, and logistical barriers [12,22,23].

The Internet Peer-2-Peer mentoring program (iP2P) for SCD adolescents builds upon previous research developing and evaluating this program for adolescents with juvenile idiopathic arthritis (JIA) and chronic pain – which both showed feasibility and preliminary clinical effectiveness [17,18]. This program emphasizes the integration of peer support within the healthcare system by matching patients (mentees) with previous patients in the clinic (mentors) to improve self-management. Mentors are trained to help guide discussions with participants, with the goal of improving management of SCD. No research has examined the feasibility and preliminary effectiveness of an online video call-based peer mentoring program for adolescents with SCD. Given the higher disease-related mortality during the transition to adult care, which is more pronounced in those with SCD, peer support can also address clinical care gaps and meet the unique needs of adolescents with SCD [13,24].

The primary objectives of this study were to determine: (1) participant accrual and withdrawal rates, (2) adherence to iP2P program and protocol, (3) acceptability of the iP2P program, (4) and adverse events. The secondary objective was to assess the preliminary effectiveness of the iP2P program compared to the control group at improving pain, anxiety, depression, self-management, and social support and qualitatively characterize the content of mentor–mentee conversations.

## Methods

### Trial Design

A two-arm waitlist pilot RCT design with a 1:1 allocation ratio was used to examine the feasibility, acceptability, and effectiveness of the iPeer2Peer program in adolescents with SCD. The trial was registered on ClinicalTrials.gov (NCT01572896). Research ethics board approvals were obtained from Clinical Trials Ontario [REB #1727] (for The Hospital for Sick Children [SickKids] and Children’s Hospital for Eastern Ontario [CHEO]) and Jim Pattison Children’s Hospital (REB #1387).

### Participants

Adolescents with SCD were recruited from four tertiary care pediatric hospitals, three in Canada, and one referral site in the USA, between January 2020 and August 2022. Adolescents were included in the study if they were: (a) aged 12–18 years, (b) diagnosed with SCD by a hematologist, (c) able to speak and read English, (d) had access to Internet connection with computer capable of using free Skype software, and (e) willing and able to complete online measures. Adolescents were excluded if they: (a) had significant cognitive impairments, (b) major co-morbid illnesses (medical or psychiatric conditions) likely to influence HRQL assessment, or (c) were currently participating in other peer support or self-management interventions.

Adolescents were recruited through two methods: (1) in-person recruitment in clinic and (2) study information letters. In the first method, local research staff screened hematology clinic schedules to identify eligible adolescents. During their scheduled visit, research staff contacted a healthcare provider in their circle of care who introduced the study to them. Interested eligible participants were provided detailed study information, and informed consent was obtained by the local research staff. The second method involved mailing potentially eligible adolescents study information letters prior to their scheduled appointment, after which research staff followed up via telephone and obtained informed consent. In response to the COVID-19 pandemic, most in-person clinic visits shifted to virtual platforms, and as such recruitment was completed through study information letters, telephone contact, and informed consent obtained remotely through Research Electronic Data Capture (REDCap). For adolescents unable to consent for themselves, their caregivers provided informed consent, and assent was obtained from the adolescent.

### Interventions

#### Experimental group

Those in the experimental group received the iPeer2Peer SCD program in addition to standard medical care. The virtual peer mentorship program consists of up to 10 sessions of approximately 30 min video calls over 15 weeks. Designated team member listened to each audio file within 48 h to ensure both participant safety and adherence to the established iPeer2Peer framework. Mentoring sessions occurred only during the scheduled times and mentee–mentor contact outside of the sessions was discouraged. More information about the program and its development were previously published [17,18].

#### Waitlist control group

Adolescents with SCD in the control group received usual care provided at their clinic for the 15 weeks course of the study. After completion of study questionnaires at 15 weeks, the adolescents in this arm were offered the iPeer2Peer SCD program.

#### Peer mentor selection

Peer mentors were young adults living with SCD who had successfully transitioned to adult care. Five to seven individuals were nominated by the healthcare teams at each institution and were screened for interest and eligibility using the following inclusion criteria: (1) between the ages of 19 and 25 years, (2) diagnosed with SCD, (3) nominated by a member of their healthcare team as a good potential mentor, (4) self-reported adherence to current treatment plan (80–100% compliance), (5) self-reported successful transitioning to an adult hematologist, (6) no active psychological disorder or a stable psychological disorder and followed by a physician/psychologist/psychiatrist, (7) self-reported self-efficacy in their ability to manage their SCD-related symptoms, (8) willingness to commit to training and mentoring participants, and (9) good communication skills as per the discretion of nominating healthcare team. Additionally, previous experience in a professional environment (e.g., as a camp counselor, part time job, and volunteering) was an asset.

#### Peer mentor training

**Figure 1.**
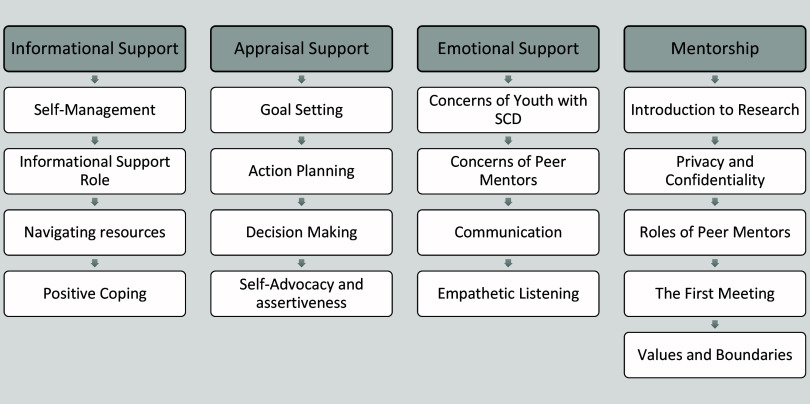
Topics reviewed during peer mentor training. SCD = sickle cell disease.

Prior to beginning the program, all peer mentors that were interested and passed the screening process completed a 2.5-day training (20 h total) program. The training covered topics listed in Figure 1 and were delivered as lectures, active group discussion, case examples, small group activities, and role play activities. In addition, mentors received standardized training protocol which included all training materials as well as additional resources and reading lists. Mentors received gift cards for their participation in this study.

### Outcomes

Outcome data consisted of participant self-reported questionnaires, measured at two time points: baseline (T1; after consent; before randomization) and after program completion (T2; 15 weeks after randomization). All questionnaires were completed online through the secure web-based system REDCap hosted at the Hospital for Sick Children.

### Demographic and baseline characteristics

Demographic and baseline characteristics were measured using an investigator-developed questionnaire which captured participant sociodemographic.

### Primary feasibility outcomes

#### Accrual

Accrual rates were calculated from study tracking logs. Accrual was calculated as those consented over those approached and those randomized over those consented.

#### Withdrawal rate

Withdrawal rates were calculated from study tracking logs. Withdrawal was calculated as those lost to follow-up (i.e., those who did not complete the study questionnaire at T2) over those randomized.

#### Adherence to iP2P program

Adherence rates were calculated from study tracking logs. Adherence was calculated and those who completed 10 weekly calls over the 15-week period over those randomized to the intervention group.

#### Adherence to study protocol

Adherence rates were calculated from REDCap logs. Adherence was calculated as those who completed all questionnaires at T1 and T2 over those randomized and by allocation group.

#### Acceptability

Acceptability of the program was set to be measured through an optional semi-structured phone interview with adolescents who received the iPeer2Peer program. However, none of the participants responded to our emails and calls, and this may be a result of the potentially long duration between the end of the study and the follow-up interview. Acceptability of the program was then measured through the mentor feedback calls after the program was completed.

#### Adverse events

Unanticipated problems and adverse events were tracked and reported from study tracking logs.

### Secondary effectiveness outcomes

To assess the preliminary effectiveness of the iP2P program compared to waitlist standard care, the following outcomes were assessed using validated questionnaires: pain intensity, pain interference, pain burden, anxiety, depression, self-management, and social support (see Table 1 for details). These were selected based on a systematic review of peer support interventions in youth in chronic disease [16].

**Table 1.** Effectiveness outcome measures

Outcome	Measurement tool	Description
Anxiety	PROMIS Pediatric Anxiety – Short Form [44,45]	A 8-item scale used to measure anxiety symptoms in pediatric populations. rated using a 5-point Likert scale ranging from 1 (“*never”*) to 5 (“*almost always”*). Total raw scores are transformed to a standardized T-score (population mean = 10, standard deviation = 10), with higher scores suggesting greater anxiety symptoms. Psychometrics: Item Response Theory was used to develop this tool, validated in adolescents with sickle cell disease pediatric and other pediatric pain populations.
Depression	PROMIS Pediatric Depression – Short Form [44–46]	A 8-item scale used to measure depressive symptoms in pediatric populations. rated using a 5-point Likert scale ranging from 1 (“*never”*) to 5 (“*almost always”*). Total raw scores are transformed to a standardized T-score (population mean = 10, standard deviation = 10), with higher scores suggesting greater depressive symptoms. Psychometrics: Item Response Theory was used to develop this tool, validated in adolescents with sickle cell disease pediatric and other pediatric pain populations.
Pain Intensity	PROMIS Pediatric Pain Intensity – Short Form [45,47]	A single item used to self-report pain average pain in the past 7 days from no pain (0) to worst pain (10). Total raw scores are used with higher scores suggesting greater pain intensity. Psychometrics: evidence of reliability (Cronbach’s alpha = 0.82) and convergent validity. This tool has been used in pediatric pain populations, including SCD.
Pain Interference	PROMIS Pediatric Pain Interference – Short Form [45,48]	A 8-item scale used to measure self-reported difficulties in completing daily activities, socioemotional problems, and impairment in physical functioning due to pain among pediatric populations with chronic conditions, rated using a 5-point Likert scale ranging from 1 (“*never”*) to 5 (“*almost always”*). Total raw scores are transformed to a standardized T-score (population mean = 10, standard deviation = 10), with higher scores suggesting greater pain interference. Psychometrics: Item Response Theory was used to develop this tool, validated in adolescents with sickle cell disease pediatric and other pediatric pain populations.
Pain Burden	Sickle Cell Disease Pain Burden Interview – Youth [6]	A 7-item scale used to measure self-reported impact of pain on physical, social/community, and emotional aspects of daily function on a 5-point Likert scale ranging from 0 “none” to 4 “every.” Psychometrics: Strong internal consistency reliability in both outpatient (α = 0.909) and inpatient groups (α = 0.821). Strong test–retest reliability after 1 week (*r =*0.80, *p*< 0.001) and construct and discriminant validity.
Perceived social support	PROMIS Pediatric Peer Relationships [49]	A 8-item scale used to measure the quality of self-reported peer relationships. Items are rated using a 5-point Likert scale ranging from 1 (“*never”*) to 5 (“*almost always”*). Total raw scores are transformed to a standardized T-score (population mean = 10, standard deviation = 10), with higher scores suggesting better peer relationships. Psychometrics: Item Response Theory was used to develop this tool, good test–retest reliability (ICC = 0.81), internal consistency (α = 0.83–0.95), good construct validity, and responsiveness. This tool has also been used in pediatric pain populations.
Self-management	TRANSITION-Q [50]	A 14-item generic tool to capture self-management skills in adolescents (12–18 years old) with chronic conditions. Response options are: 2 (“*Always”*), 1 (“*Sometimes”*) or 0 (“*Never”*). Items are transformed to a 0–100 scale, with higher scores indicate greater self-management. Psychometrics: Person Separation Index = 0.82; no differential item function by age or gender; low residual correlations between items; Cronbach’s α = 0.85; test–retest reliability = 0.90. This tool has also been used in pediatric pain populations.

PROMIS = Patient-Reported Outcomes Measurement Information System.

### Secondary qualitative outcomes

To characterize the content of mentor–mentee conversations, a qualitative analysis was completed using transcribed audio-recorded of mentor–mentee calls based on levels of engagement.

### Sample Size

We aimed to recruit 40 participants, 20 in in each arm. This is in line with Hertzog suggestion of 20 to 30 participants per group for pilot feasibility studies [25].

### Randomization

Following completion of baseline questionnaires, participants were allocated to the intervention or control arm using Randomizer, a software application that manages randomization of participants in clinical research. Randomization was centrally controlled and concealed.

### Blinding

Healthcare providers and study investigators were blinded to group allocation, while participants and research staff were not.

### Quantitative analysis

Data were analyzed using STATA version 15.1. Demographic and baseline characteristics were summarized as mean and standard deviation for continuous outcomes and raw count and percentages for categorical outcomes for each allocation group. Rates of accrual, dropout, compliance, and missing data were calculated and reported descriptively. All available data based on participant’s allocation group was used to analyze the secondary effectiveness outcomes. Linear mixed effects models were used to assess the intervention effect, with time, group, and an interaction by time and group as predictors.

### Qualitative analysis

Seventy five calls that represent 12 complete mentor–mentee pairings were transcribed verbatim and analyzed using deductive and inductive content analysis [26]. Using all 75 conversations allowed for both rich and thick descriptions of the topics of mentorship–mentee conversations given the small sample size. The calls were divided by level of engagement for further analysis. Level of engagement was categorized into three groups based on the number of calls each mentee completed. The low group (*n* = 3 mentor–mentee dyads) consisted of mentees who had 1–3 calls, moderate (*n* = 2 mentor–mentee dyads) 4–6 calls, and high 7–10 (*n* = 7 mentor–mentee dyads) calls. Initially, two investigators (B.W. and L.K.) leveraged an inductive open coding approach to develop an initial coding framework with categories and sub-categories, drawing from the coding structures of published iPeer2Peer pilot and feasibility trials. Next, the same investigators reviewed three transcripts independently and met to discuss and develop codes. Codes represent meaningful units (phrases to several sentences) which address research question. Three transcripts were independently coded using the developed codes, and two investigators met again to discuss and refine the coding structure. Next, four team members (B.W., E.T., L.K., and S.O.) independently coded all the remaining transcripts, consistently communicating to ensure consensus and address any emerging codes. If new codes arose, a comprehensive review of all previous transcripts was conducted to incorporate these additions. Finally, the original two investigators (B.W. and L.K.) met to compare and confirm that all the previously developed categories and sub-categories aligned with the initial coding framework. The percentages of each code that arose from grouping together codes during coding, and these were evaluated to determine and separate the most prominent codes for each level of engagement. All analyses were completed on Dedoose [27].

## Results

### Participants

**Figure 2.**
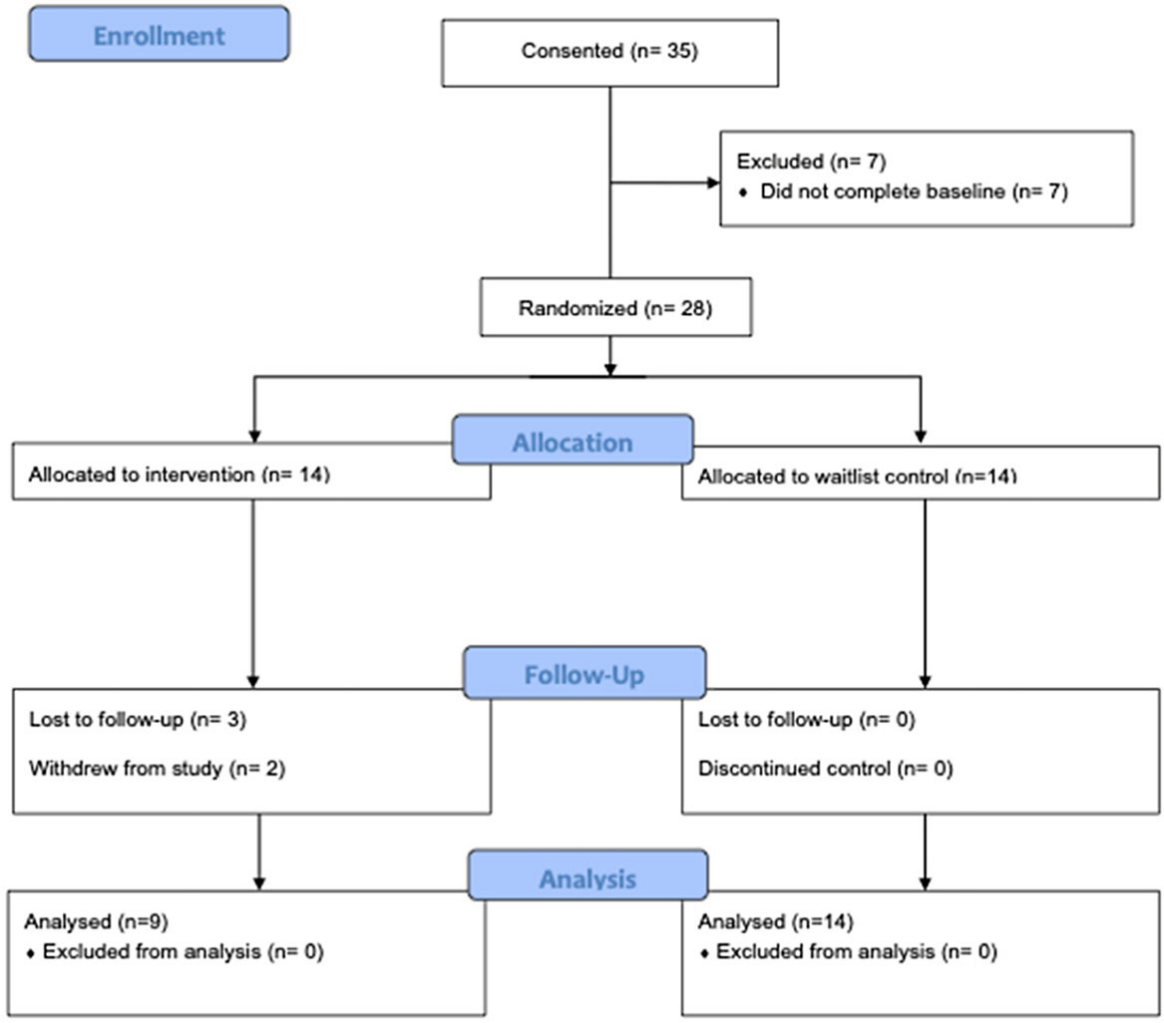
Consolidated standards of reporting trials flow diagram. *Note:* Screening and eligibility data collected during recruitment was not available from study sites. As a result, it is not provided in the flow diagram.

Participant enrollment began in January 2020 and ended August 2022, with final follow up completed in December 2022. The Consolidated Standards of Reporting Trials (CONSORT) extension to randomized pilot and feasibility trials flow diagram is presented in Figure 2. Since lower-than-expected recruitment rates were achieved, the study closed at the end of planned recruitment period. Participant demographic and baseline characteristics are presented in Table 2. Most mentees were female (16/28, 57%), aged 12–18 years, with an average age of 14.8 years (SD 1.7), identified as Black (25/28, 89%), predominately had the Hemoglobin SS genotype (19/28, 68%), and were currently taking hydroxyurea (23/28, 89%).

**Table 2.** Adolescent demographic and disease characteristics

	Intervention (*n* = 14)	Control (*n* = 14)	Total (*n* = 28)
Age in years, mean (SD), median, range	14.5 (1.81), 14.2, 12–17	15.1 (1.67), 14.7, 12–18	14.8 (1.7), 14.4, 12–18
Gender, *n* (%)			
* Girl/Woman*	10 (72)	6 (43)	16 (57)
* Boy/Man*	4 (28)	8 (57)	12 (43)
Race			
* Black*	13 (93)	12 (86)	25 (89)
* White*	1 (7)	0	1 (4)
* Mixed Race*	0	2 (14)	2 (7)
SCD Genotype, *n* (%)			
* Hemoglobin SS*	9 (64)	10 (72)	19 (68)
* Hemoglobin SC*	3 (22)	2 (14)	5 (18)
* Hemoglobin S beta-thalassemia zero*	2 (14)	0	2 (7)
*I do not know*	0	2 (14)	2 (7)
Currently taking hydroxyurea, *n* (%)	10 (77)	13 (93)	23 (89)

### Primary outcomes

#### Accrual

Thirty-five participants were consented over 31 months. The accrual rate calculated as those consented over those approached could not be calculated as the tracking logs were not maintained by all subsites, as such the number approached could not be determined. Additionally, the shift to remote recruitment as a response to the COVID-19 pandemic resulted in more letters and phone calls, but few responded to those letters or calls. Accrual, measured as those randomized over those consented was 80% (28/35).

#### Withdrawal rate

Calculated as those lost to follow-up (i.e., those who did not complete the study questionnaire at T2) over those randomized was 18% (5/28). Reasons for withdrawal were mostly due to a loss of interest in the study.

#### Adherence to IP2P program

Calculated as those who completed 10 weekly calls over the 15-week period was 28% (4/14) among those randomized into the intervention. Overall, 71% (10/14) of adolescents randomized to the intervention completed at least one call, completing on average 6 calls, and these calls typically lasted 28.0 (SD 16.1; range 7–121) min. Among the four male participants who were randomized, only one completed calls (total 7) with their mentor. The participants randomized into the control group were offered the iP2P program after the 15-week study period, 2/14 (14%) participants completed calls with a mentor, and none reached 10 weekly calls.

#### Adhere to study protocol

Calculated as those who completed all questionnaires at T1 and T2 was 71% (20/28) for all participants, 50% (7/14) among the intervention group, and 93% (13/14) among the control group. Three participants partially completed the questionnaires, as such adherence measured as partial completion of questionnaires was 82% (23/28). Table 2 reports the adherence for each questionnaire.

#### Acceptability

Three mentors were interviewed after completing the program – during these conversations they highlighted their positive experience with the program. Outlining shared experiences and navigating adolescence with chronic pain as common themes explored in mentor–mentee conversations. Key feedback about the program included seeking more structure, reduced time between initial mentorship training, and assigning mentees, which was often sporadic. Providing prompts and guidelines about conversations and the need for more male mentors in the mentor pool were also suggested. Mentors indicated that mentees were engaged with the program and were more assertive and comfortable with SCD by the end of the required sessions. Finally, mentors were motivated to participate in the study as they were lacking a similar resource during their adolescence. All mentors reported willingness to recommend this experience to peers living with SCD as it allowed them to showcase their growth while living with chronic pain.

#### Adverse events

No adverse events were reported by participants.

### Quantitative results – secondary effectiveness outcomes

Due to high withdrawal rates among participants randomized into the intervention group, we were unable to report planned secondary analyses. Instead, these are descriptively presented in Table 3.

**Table 3.** Secondary effectiveness outcomes at baseline and end point

	Intervention *N* = 14				Waitlist control *N* = 14			
	Baseline		Endpoint		Baseline		Endpoint	
Measure	*N*	Mean (SD)	*N*	Mean (SD)	*N*	Mean (SD)	*N*	Mean (SD)
Transition-Q	13	46.61 (11.2)	7	57.00 (9.93)	14	57.78 (10.3)	14	59.07 (8.84)
PROMIS Pain Interference	14	59.07 (12.9)	9	59.11 (12.2)	14	59.96 (6.06)	14	58.65 (12.1)
PROMIS Depression	14	50.53 (10.7)	8	49.67 (10.4)	14	51.35 (7.51)	14	52.72 (8.42)
PROMIS Anxiety	14	48.91 (8.27)	9	47.15 (10.8)	13	50.19 (12.2)	14	50.9 (12.6)
PROMIS Pain Intensity	14	3.07 (2.61)	9	3.66 (3.12)	14	2.78 (3.19)	14	2.92 (2.97)
Sickle Cell Disease Pain Burden Scale	14	6.00 (6.06)	9	3.22 (3.56)	13	5.23 (7.75)	13	3.30 (5.37)
PROMIS Peer Relationships	13	40.87 (6.51)	9	43.3 (4.59)	14	44.42 (7.72)	9	48.07 (6.29)

PROMIS: Patient-Reported Outcomes Measurement Information System.

### Qualitative results

#### Call content

From an analysis of 75 transcripts, three distinct categories emerged: Impact of SCD, Self-Management, and Transitioning to Adulthood with SCD. An additional category, General Topics, captures discussions on miscellaneous or non-SCD-related items. Table 4 contains category- and sub-category-specific quotations, and Table 5 contains a percentage distribution of each category and sub-category across all mentor–mentee calls.

**Table 4.** Call content and supportive quotations of the experiences of mentors and mentees with sickle cell disease who participated in the iPeer2Peer program

Mentee/ Mentor	Category	Quote
Mentor	Disease Management	*“Thank you so much for letting me be your, be your guide through this whole sickle cell thing…And also I just want you to know you’re not alone in the world. You are truly not alone in the world. There’s so many people suffering from this disease…So be grateful and enjoy life. You know life is too short to you know, to be… to be down over small things. Life is, I’m telling you from the 21 years of experience I’ve had on this planet, life is a rollercoaster it’s all ups and downs, ups and downs, but when you’re there, you know the ups, just be thankful have fun and be thankful to God that you are in the ups.”*
Mentee – Female, 13 years, high engagement	Mentee/Mentor interests	*“Yeah, I find this really fun, especially well like you said, we have like quite a few similarities too, so that was really cool and it was not scary. The first time I was really scared but after a while I’m like oh yay. I guess I would recommend it to someone, especially like, uh, I do not know how to… I do not know how to say this. Uh, yeah I would recommend it to someone, if they rather call someone if they’re happy with the person there, I guess it just depends on the person, if you are in college, and maybe you might not like them or might not like it as much. But yeah. I found this fun.”*
Mentor	Self-advocacy	*“Last week you asked me what do I tell people about my sickle cell and I’m like “oh I don’t tell people’ and I realized that was a lie it’s like the first thing that comes up. Like in like job interviews I’m like yeah I have sickle cell so I might be gone for a week or 2 a month.”*
Mentee – Female, 17 years, medium engagement	Daily Life Experiences	*“It was so many grade 9. I did the same thing in grade 9. ‘cause my first year wasn’t that like. Well, not like peer pressure. You know all that stuff. That didn’t happen. I still had my middle school friends and like green line. So like we still read together and like I read a lot of books for grade 10 hit. New friends, new interests and bye bye yeah I feel bad for ditching my old friends at the same time like their personality did not fit mine anymore. It was just awkward just hanging out. I mean, it’s sad, but it’s like to happen.”*
Mentor	Health Habits	*“I’m gonna ask you some questions about balanced diets, how, how, how well do you eat like do you eat healthy stuff like in your family? Do you eat healthy or you do healthy and a little bit of unhealthy or even just unhealthy”*

**Table 5.** Distribution of call content by engagement category

	Low engagement	Moderate engagement	High engagement
**Impact of sickle cell disease**	**29%**	**15%**	**18%**
Extracurricular activities	0%	0%	1%
Jobs/employment	2%	1%	1%
Mental and emotional health	6%	2%	2%
Perceived self-efficacy	3%	1%	2%
Physical wellness	7%	5%	5%
Relationships	7%	3%	4%
School/academics	4%	4%	2%
**General topics**	**24%**	**47%**	**52%**
Clinical/medical visits	1%	1%	2%
COVID-19 pandemic	0%	7%	3%
Daily life experiences	8%	18%	18%
Family and friends	2%	5%	9%
Mentee/mentor interests	7%	8%	10%
Non-disease-related adolescent issues	5%	4%	7%
Technical or logistic issues	3%	4%	2%
**Self-management**	**20%**	**15%**	**15%**
Coping mechanisms	4%	3%	2%
Disease management	12%	6%	6%
Goal setting	2%	2%	2%
Health habits	3%	5%	5%
**Transition to adulthood with sickle cell disease**	**26%**	**23%**	**15%**
Awareness and use of available resources	3%	2%	2%
Education, knowledge acquisition, or skill development	5%	5%	3%
Health system navigation	5%	5%	4%
Navigating systemic barriers and/or marginalization	1%	3%	1%
Recognizing needs	7%	4%	3%
Self-advocacy	5%	4%	2%
**Total**	**100%**	**100%**	**100%**

#### Impact of SCD

This category covers the holistic impact of SCD on various aspects of an adolescents’ life. These include conversations on the effects of the disease on interpersonal relationships, mental and emotional well-being, academic pursuits, perceived self-efficacy, employment, physical wellness, and engagement in extracurricular activities. This category consistently garnered attention across all engagement levels, with a substantial focus (29%) in the low engagement group, indicating a prevalent need for discussions on various aspects of life affected by SCD. While the moderate (15%) and high (18%) engagement groups suggest a potential broadening of topics beyond the impacts of SCD.

#### Self-management

The category encompasses discussions on coping mechanisms, disease management, health habits, and goal setting. It provides a platform for mentors and mentees to navigate the challenges associated with SCD by sharing strategies for physical, emotional, and psychological well-being, adhering to medical treatments, adopting healthy habits, and setting and achieving personal goals despite health challenges. Topics relating to self-management were most often discussed in the low engagement group (20%), and less so in the moderate (15%) and high (15%) groups.

#### Transitioning to adulthood with SCD

This category encompasses a range of discussions centered around preparing adolescents with SCD for the challenges and opportunities of adulthood. Mentor–mentees had discussions on acquiring knowledge and skills related to living with SCD, advocating for oneself within healthcare and institutional settings, navigating the healthcare system, recognizing personal needs, experiences with managing systemic barriers and marginalization, and identifying and utilizing resources for support. These discussions were geared toward equipping mentees with the tools and awareness needed to transition successfully into adulthood while managing the challenges posed by SCD. This category consistently dominates discussions across all engagement levels, representing 26 and 23% of call content focus in the low and moderate engagement groups, respectively, while representing (15%) in the high engagement group.

#### General topics

This category encompasses a wide array of topics beyond the immediate impact of SCD, offering a comprehensive view of mentor–mentee interactions. General topics and miscellaneous items constitute 24% of the total call content for low engagement pairs. Moderate engagement pairs witness a substantial increase in the “General Topics” category (47%), reflecting a broadened conversation spectrum, with the COVID-19 pandemic (7%) and family and friends (5%) gaining significance. High engagement pairs continue this trend, with the “General Topics” category dominating (52%). The consistent presence of technical or logistic issues (2–4%) across engagement levels underscores the ongoing need for programmatic support in overcoming practical challenges.

### Mentor–mentee engagement and call content

#### Low engagement

The low engagement group (1–3 video calls) consisted of three mentor–mentee dyads, with a marked emphasis on the “Impact of SCD” category, constituting 29% of call content, highlighting immediate concerns faced by mentees. Within this, the impacts of SCD on physical wellness (7%), relationships (7%), and mental and emotional health (6%) emerge as dominant sub-categories, signifying a focus on tangible impacts of the disease and interpersonal dynamics. Notably, disease management covers 12% of all call content in this group. Comparing across engagement groups, disease management is notably more prevalent in low engagement pairs than in moderate (6%) and high (6%) engagement pairs. Suggesting even with limited interactions, low engagement pairs prioritize discussions on practical aspects of managing SCD, possibly indicating a heightened awareness of the importance of self-management strategies. Conversely, the impact of SCD on extracurricular activities represent the least discussed (0% of call content) sub-category in this group. Within the “Transition to Adulthood with SCD” category, the sub-category of “Recognizing Needs” constitutes 7% of call content in these pairs, indicating a significant focus on discussions about recognizing one’s own needs and limitations related to SCD. Comparatively, “Recognizing Needs” comprises 4% of call content in moderate engagement pairs and the 3% in high engagement pairs. In the “General Topics” category, technical or logistic issues receive minimal attention at 3%, suggesting a potential gap in addressing practical program-related challenges within low engagement pairs.

#### Moderate engagement

The moderate engagement group (4–6 video calls) consisted of two mentor–mentee dyads, with a sustained significance of the “Impact of SCD” category at 15%. The “General Topics” category at 47%, with discussions on family and friends increasing to 9%, indicating a widening scope of conversation topics. While disease management in the “Self-management” category retains importance at 6%, the coverage diminishes compared to the low engagement group, suggesting a subtle shift in focus. In comparison with the low engagement group, moderate engagement pairs explore a more varied range of topics within disease management. Notably, discussions on coping mechanisms (3%) were reduced.

#### High engagement

In the high engagement group (7–10 video calls) consisted of seven mentor–mentee dyads. This group also showed a sustained focus on the “Impact of SCD” category at 18% underscoring the importance of exploring the impact of SCD, such as daily life experiences (18%) and relationships (4%). In the “Self-management” category, disease management remains a crucial sub-category, covering 6% of all call content. While coverage of disease management is lower compared to the low engagement group, it reflects a sustained focus on the practical aspects of managing SCD. Moreover, within the “Transition to Adulthood with SCD” category, the sub-category of education, knowledge acquisition, or skill development (3%) was decreased, potentially indicating a reduced emphasis on education and learning-specific discussions within these pairs. In the “General Topics” category, family and friends discussions (9%) and mentee/mentor interests (10%) were dominant, a notable increase compared to the low engagement group. These nuanced shifts suggest that high engagement pairs foster diverse discussions, incorporating personal interests, family dynamics, and broader adolescent experiences while maintaining a balanced approach to disease management discussions.

## Discussion

Given the proposed benefits of online peer support in improving self-management, our study assessed the feasibility of the iP2P program in adolescents with SCD [17,18]. Despite this, the results from our pilot study indicate the program is unfeasible due to: low adherence and engagement, recruitment challenges due to the pandemic, and low outcome completion rates, preventing us from completing the proposed analysis to determine the effectiveness of the program. This contrasts with the pilot studies of the iP2P program in youth with JIA and chronic pain, which showed feasibility and preliminary clinical effectiveness [17,18]. These differences may be a result of the unique clinical needs of adolescents with SCD compared to other chronic pain conditions, such as frequent pain crises or greater reliance on acute care settings [3,28]. Furthermore, adolescents in this study were racialized, which may compound their experiences of health inequities, including barriers to healthcare access, socioeconomic challenges, and systemic discrimination [12,29,30].

The qualitative results suggest that participants who were engaged enjoyed the program and were able to deepen their understanding of SCD and learn new ways to improve self-management. Similar to iP2P JIA, most of the SCD mentees did not report high pain intensity, and this may have resulted in the shift to non-disease-related topics and could explain the low adherence and engagement with the program [18]. Adolescents with higher pain may benefit most from this program; however, these same individuals may be difficult to engage or are not able to access iP2P due to their pain. Mentors who were engaged also had a positive experience, finding gratification in being a resource to mentees in a way that was never available to them when they were younger. Mentors also recommended including more prompts to guide discussion and more male mentors. Recognizing each participant’s distinct pain characteristics, future program iterations can include training modules that teach mentors to prepare calls based on the needs of their mentees, as well as explore ways to support individuals with higher needs, as implemented in the iP2P JIA study [18].

A discernible shift in conversation is observed from personal struggles and immediate impacts of SCD in low engagement pairs, to more diversified and proactive discussions in highly engaged pairs. Daily life experiences, family and friends, and non-disease-related adolescent issues become more prevalent, emphasizing a holistic approach to mentorship that encompasses various aspects of mentees’ lives. While certain themes such as disease management and health habits remain consistently discussed across all engagement levels, there is a noticeable decrease in discussions related to coping mechanisms, impact of SCD on relationships, recognizing needs, and self-advocacy in highly engaged pairs. Suggesting potential shift toward more proactive approaches in managing challenges, diversifying conversation topics, and fostering increased self-sufficiency and resilience.

As engagement intensifies, a broader range of topics beyond the immediate impact of the disease are discussed, fostering a more meaningful relationship as the mentor–mentee spent more time together compared to the low engagement group. Perhaps these mentees were more willing to have weekly conversations as they were more interested in creating a “friendship,” looking to gain more from the program and were willing to be open during conversations. In a study that conducted a focus group with adolescents and parents to gather important themes for the development of a peer support group, most of the adolescents mentioned these aforementioned factors [31]. Moreover, they reported that peer support programs would be beneficial after diagnosis or during critical times and should be voluntarily, and both mentors and mentees should be invested [31]. Interestingly, the same mentors were consistently in the highly engaged dyads. These individuals were enthusiastic about the iP2P program, had outgoing personalities, prepared for mentoring sessions with questions and discussion points, and were responsive to the research team. Although our sample size for mentors was small, these qualities may have facilitated a stronger connection with their mentees.

Research has found differences in engagement with different technology-based peer support interventions, based on the adolescent preferences [23]. The iP2P program only provided a video call option, and this could have attributed to the level of engagement; some adolescents may prefer instant messaging instead. Studies that have assessed the use of daily messaging and app interventions found high satisfaction, suggesting that adolescents may have higher tolerance for this type of communication [32–34]. Moreover, text messaging might also be an easier mode of communication between mentees and mentors, given high rates of hospitalizations among individuals with SCD [35]. Furthermore, research across various clinical populations suggest that sociodemographic and cultural factors can reduce rates of uptake and engagement with digital health interventions – adolescents with SCD experience many of these same factors [36–38]. Future iterations of the program could directly include training material that recognizes stress from minority status or limited resources, incorporate culturally tailored strategies to manage these stressors (e.g., emphasizing empowerment), and recognize the possible mistrust of research in these communities, alleviating this through cultural sensitivity training of research staff and mentors [39–41].

It is evident that besides “general life” topics, mentees also wanted to discuss other topics such as academics and relationships. The emotional toll of SCD may result in adolescents perceiving their life and suffering as unfair. One study described the pain-related injustices that youth with SCD face and how these contribute to worsening pain, anxiety, and depression [42]. Since SCD is a condition that is chronic, it is important for adolescents to navigate these negative thoughts early on, so the effect of SCD can be reduced and self-management can be better. Peer support offers a useful platform to discuss these challenges with individuals with similar disease and social experiences.

Limitations of this study include the small size due to recruitment challenges, low adherence, and engagement. Given the small sample size and retention, we were not able to complete proposed secondary effectiveness analysis which may have given us a better indication of the effect of the program. Gender may also influence adherence and engagement as males may prefer a different style of mentoring than those offered or preferred same-gender pairings, as found in previous iP2P studies [18,43]. Future iP2P programs can ensure there is an equal balance of genders with the mentors and offer alternative modes of communication. Furthermore, we were unable to explore potential age-related differences as most participants were 13–16 years old, limiting the generalizability of the study to younger teens. Finally, additional clinical (e.g., frequency of pain crisis and medication adherence) and socioeconomic (e.g., household income and parental employment status) factors would have been helpful to contextualize feasibility and acceptability; however, these data were not collected. Future research should collect boarder demographic and clinical characteristic data to facilitate such exploration.

## Conclusion

This study explored the level of engagement, satisfaction, and topics of conversation during the iP2P program for adolescents with SCD. For those able to engage, the program was beneficial in allowing adolescents to use their mentors as resources regarding disease and non-disease-related topics, covering the many struggles’ adolescents face which is a key platform to have that complements standard care. Modifications and future research with the iP2P program can focus on implementation/access via personalized mentoring, flexibility in communication, and a stronger focus on gender, which can all help refine this platform to provide the necessary support adolescents with chronic illness need.

## Data Availability

The raw data will not be made available in order to protect against the possible identification of any patients who took part in the study. As per institution REB, any release of data requires contracts outlining the recipient of data and purposed use of data.
